# Social media usage and cyberbullying: the moderating role of tie strength

**DOI:** 10.3389/fpsyg.2025.1490022

**Published:** 2025-02-11

**Authors:** Jinru Ni, Hongyu Fu, Yajing Zhu, Zewen Li, Shuyi Wang, Haoran Su

**Affiliations:** ^1^School of Journalism and Communication, Guangzhou University, Guangzhou, China; ^2^School of Education, Guangzhou University, Guangzhou, China; ^3^School of Economics and Statistics, Guangzhou University, Guangzhou, China; ^4^School of Mathematics and Information, Guangzhou University, Guangzhou, China

**Keywords:** media usage, cyberbullying, tie strength, medium theory, moderate effect

## Abstract

**Introduction:**

Cyberbullying is a pervasive issue in the digital age, closely linked to social media usage. However, existing research has largely overlooked the role of tie strength on social media platforms in shaping cyberbullying dynamics. This study, grounded in tie strength theory and medium theory, investigates the association between social media usage and cyberbullying, focusing on how tie strength moderates this relationship.

**Methods:**

A sample of 813 healthy adults (M_age_ = 20.06 ± 2.30 years, 498 females) completed an online survey, including the Chinese version of the Social Network Site Intensity Scale and the Cyberbullying Inventory for College Students (CICS).

**Results:**

(1) Tie strength varies from platform to platform. The order of tie strength between users and the four platforms is as follows: WeChat > Bilibili > Weibo > Douyin. (2) Strong-tie social media platforms exhibited higher levels of users’social media engagement compared to those with weak ties. (3) Weak ties significantly moderated the relationship between social media usage and cyberbullying, whereas strong ties did not. Increased social media usage was associated with a higher likelihood of both engaging in and being a victim of cyberbullying on weak-tie platforms, while strong-tie platforms showed a lower likelihood of both engaging in or being a victim of cyberbullying with increased usage.

**Conclusion:**

These findings highlight the interplay between tie strength theory and medium theory in explaining cyberbullying dynamics and underscore the need for platform-specific interventions to address this pervasive issue.

## 1 Introduction

Cyberbullying is the intentional and persistent use of electronic technologies by individuals or groups to harass and victimize others (Kowalski and Limber, 2013). The widespread availability of internet technology has allowed online bullying to transcend the limitations of the physical environment. This makes cyberbullying a widespread social problem (Kowalski et al., 2022). The core element shared by both cyberbullying and traditional bullying is the intentional aggressive behavior of the bully toward the victim. Nevertheless, cyberbullying possesses distinct characteristics that set it apart from traditional bullying (Liang et al., 2024). For one, the potential reach of cyberbullying is far broader, as the internet allows perpetrators to target victims beyond their immediate physical proximity. Additionally, assessing the degree of victimization can be more difficult in the online realm. Perpetrators of cyberbullying also frequently enjoy anonymity, which traditional bullies typically do not. Moreover, the complexity of cyber technology complicates the monitoring and management of such harmful behavior, making it challenging to identify those responsible (Wang et al., 2023).

Cyberbullying poses a great threat to one’s well-being. Both the perpetrators and victims of cyberbullying exhibit significantly higher levels of depression compared to people without any experience of cyberbullying (Tabares et al., 2024). Particularly, victims of cyberbullying tend to exhibit even stronger suicidal tendencies than perpetrators (Mui et al., 2023). Individuals who have experienced cyberbullying, regardless of their role as a bully or victim, tend to exhibit lower self-esteem than their peers who have not experienced such online harassment (Xia et al., 2023). In addition, research suggests that the more experiences of cyberbullying one has, the higher their stress symptoms (Brailovskaia et al., 2023).

Consequently, cyberbullying is a serious issue. It is imperative to conduct a comprehensive investigation into the phenomenon of cyberbullying. Such studies can enhance our understanding of cyberbullying and facilitate the development and implementation of effective prevention strategies. Moreover, these investigations can draw social attention to this issue and raise public awareness of the prevalence and impact of cyberbullying.

## 2 Literature review and hypothesis development

### 2.1 Social tie strength and users’ media usage

Interpersonal networks can be divided into strong-tie networks and weak-tie networks. Weak-tie network refers to interpersonal connections characterized by infrequent interactions and minimal emotional investment, while a strong-tie network is characterized by frequent interactions and a higher level of emotional closeness (Granovetter, 1973). In contrast to weak-tie networks, strong relationships can be the source of an individual’s sense of security (Gupta et al., 2019). Tie strength has been found to influence one’s online behavior. Specifically, individuals can be more affected by strong ties when they make decisions (Sicilia et al., 2020; Mattke et al., 2020; Wang et al., 2022). For example, close friends have a greater influence on an individual’s willingness to purchase (Sicilia et al., 2020). “Likes” from close friends can even prompt users to click to learn about social media content that was not considered valuable before (Brailovskaia et al., 2023). In addition, the strength of interpersonal ties is not only in direct human-to-human communication but also in the interactions between individuals and various social media platforms.

Medium theory, proposed by Meyrowitz (1988), mainly focuses on the differences in the ways of information transmission, reception, and interaction between different media. It posits that the use of electronic media creates different media information environments, which then affect users’ behavior. Individuals are more likely to use specific media when they agree with its culture or when it meets their needs (Chacón and Retis, 2023; Schoultz et al., 2023). They are more likely to support and trust the media (C and Majumdar, 2023). Thus, individuals establish a strong connection with the media. The media culture also subtly influences individual’s attitudes and behaviors (Liu et al., 2023; Valtysson, 2022; Takhar et al., 2021). In contrast, when individuals do not agree with the media culture, they are likely to reduce or even cease their media usage. Therefore, individuals will establish a weak connection with the media, or no connection (Bright et al., 2015). In such cases, media culture has little influence on individuals’ attitudes and behaviors (Figeac and Favre, 2023).

Previous studies have not yet delved deeply into how users’ media usage behaviors impact cyberbullying activities across different platforms. Thus, the differences in the strength of interpersonal networks established on various social media platforms are often overlooked. Conclusions about cyberbullying drawn from this type of research may not be universally applicable to all situations. Therefore, this study will subdivide the strength of interpersonal relationship networks across different platforms and explore the extent to which these strengths affect the intensity of users’ media behaviors.

Tie strength influences the transmission and processing of information within social networks (Gupta et al., 2019). People often establish strong interpersonal networks on strong-tie social media to share information and resources. However, in weak-tie social media, users come from different regions, the information received and processed by users is mostly from algorithm recommendations, which makes it difficult to form in-depth interactions. As a result, the social network relationships formed on these platforms are weaker. Young people can establish weak ties with users across different regions, spaces, and communities on weak-tie social media by watching, liking, commenting, and privately chatting. The unique platform culture created by users on different social media results in diverse styles and influences. Therefore, there may be differences in users’ social media usage across platforms with different tie strengths. Thus, the following hypothesis is proposed:

*Hypothesis 1*. Tie strength of social media has a positive impact on the intensity of users’ media usage. Specifically, the intensity of users’ media usage in strong-tie social media is higher, the intensity of users’ media usage in weak-tie social media is lower.

### 2.2 Users’ media usage and cyberbullying

As of June 2024, Chinese netizens spent an average of 29.0 h per week online, 2.9 h more than in December 2023 (China Internet Network Information Center [CNNIC], 2024). Media usage shapes the context and dynamics of interpersonal communication (Flamino et al., 2023). The emergence of social media has brought interpersonal relationships and emotional interaction into the network field (Goldenberg and Willer, 2023). This has significantly transformed the way people communicate, enabling online interactions to transcend the limitations of time and space (Ali et al., 2022). Previous research has demonstrated that personalization can enhance the cognitive and emotional engagement of media users (Sundar et al., 2015). Electronic media further shapes the social landscape, thereby influencing users’ social behaviors. Media usage, whether intentional or unintentional, induces both short-term and long-term changes in interpersonal cognition, emotion, attitude, and behavior (Valkenburg et al., 2016; Hoffner and Bond, 2022). For instance, the influence of Facebook usage is more pronounced in communication with strong-tie networks, while the impact of Twitter usage is more pronounced in communication with weak-tie networks (Valenzuela et al., 2017).

However, social media platforms have the highest incidence of cyberbullying worldwide, accounting for 65% (Ipsos, 2018). Media usage has been identified as an important risk factor for both cyberbullying perpetration and victimization (Paciello et al., 2023; Kowalski et al., 2014). Longobardi et al. (2020) found that social media usage increases the likelihood of engaging in cyberbullying. Increased media usage may also lead to a greater likelihood of being cyberbullied (Rafiq and Linden, 2024). Previous studies have indicated that users are motivated by specific reasons to search for, select, and purposefully use particular media or content (Kardefelt-Winther, 2014; Boulianne et al., 2023; Hsieh et al., 2023). Adolescents with higher aggressive traits are more likely to engage selectively with violent websites, potentially further enhancing their aggressive tendencies (Chabbouh et al., 2023). Cyberbullying is a negative behavior resulting from social media use, and the intensity of users’ engagement with social media may influence this behavior. Therefore, the following hypothesis is proposed:

*Hypothesis 2a*. Users’ media usage is positively associated with cyberbullying engagement. Specifically, higher media usage causes a higher likelihood of cyberbullying engagement. Lower media usage causes a lower likelihood of cyberbullying engagement.

*Hypothesis 2b*. Users’ media usage is positively associated with the likelihood of being cyberbullied. Specifically, higher media usage causes a higher likelihood of being cyberbullied. Lower media usage causes a lower likelihood of being cyberbullied.

### 2.3 Moderating effect of tie strength

Medium theory plays an important role in understanding the influence of different social media on individuals. According to medium theory, different media create different environments and influence individual behavior. However, media usage is associated with an individual’s self-disclosure in media (Ostendorf et al., 2020). Specifically, the higher the intensity of media usage, the greater the individual’s self-disclosure on media (Md. Motaher Hossain et al., 2023). Similarly, the lower the intensity of media usage, the lower the individual’s self-disclosure on media. Md. Motaher Hossain et al. (2023) found that individuals have less trust in the outside world and exhibit a strong sense of defense, high aggression, and increased hostility toward strangers in unfamiliar and less intimate environments (Sobkin and Fedotova, 2021). Their study highlights how the degree of interpersonal intimacy and environmental familiarity can significantly shape an individual’s psychological and behavioral reactions. Sobkin and Fedotova (2021) found that prolonged exposure to unfamiliar environments makes individuals vulnerable to attacks by strangers. Without the support and security provided by known social connections and familiar routines, individuals in unfamiliar environments may experience increased stress, anxiety, and a diminished sense of control. This heightened state of vulnerability can make them more likely targets for hostile or exploitative behaviors from unfamiliar individuals they encounter in these contexts. Moreover, the research suggests that it can be exceptionally challenging for individuals to find comfort and a sense of security when immersed in unfamiliar environments (DeSmet et al., 2014). This heightened sense of discomfort and vulnerability in unfamiliar settings can further exacerbate the psychological toll, leaving individuals feeling isolated, disoriented, and bereft of the familiar coping mechanisms they would normally employ. Thus, this exacerbates the harm. The heightened sensitivity of individuals to the outside world makes them more susceptible to external influences and responses (Harnett et al., 2015).

Gini has proved that the presence of many weak connections may lead to increased exposure to hurtful actions on Facebook (Gini, 2006). However, individuals are highly self-disclosed in strong-tie media originally. This kind of interaction persists in a stable environment for a long time. Individuals tend to be less aggressive in familiar environments and less vulnerable to attacks from strangers. It has been demonstrated that tie strength matters: High-quality and close friendships reduce involvement in victimization and perpetration (Adams et al., 2005; Bollmer et al., 2005). Therefore, the degree of self-disclosure among individuals in strong-tie media has minimal influence on those who commit or experience cyberbullying. Thus, individuals who become perpetrators or victims of cyberbullying may be influenced by tie strength. Therefore, the following hypothesis is proposed:

*Hypothesis 3a*. Tie strength moderates the effect of media usage on cyberbullying. Specifically, in strong-tie social media, media usage is not highly associated with cyberbullying. In weak-tie social media, the higher the media usage, the higher the likelihood of cyberbullying engagement.

*Hypothesis 3b*. Tie strength moderates the effect of media usage on being cyberbullied. Specifically, in strong-tie social media, media usage is not highly associated with being cyberbullied. In weak-tie social media, the higher the media usage, the higher the likelihood of being a victim of cyberbullying.

### 2.4 The current study

The current study aims to investigate the association between social media usage and cyberbullying, while further testing the moderating role of tie strength across various social media platforms in this relationship. We measured participants’ social media usage intensity and their experiences of cyberbullying across four major social media platforms: WeChat, Weibo, Douyin, and Bilibili. WeChat is an instant messaging application. Users can only start chatting if they have authenticated each other as WeChat friends. Additionally, users can post their latest updates and keep up with their friends’ news in Moments. It is deeply integrated into the daily lives of Chinese citizens and offers functions such as instant messaging, ecommerce, and dynamic content sharing. This allows users to closely communicate with their inner circle, consolidating strong interpersonal ties and relationships. Consequently, individuals establish a strong-tie social network on WeChat. Weibo is an information-sharing and communication platform that provides entertainment, leisure, and life services for the public. Users can post content, repost, comment, search, or follow other users on Weibo. Douyin is a short video software with a huge user base. It uses big data technology to recommend videos that match users’ preferences. Users can like, comment, share videos, create and post videos. Bilibili is a video platform for content creation and sharing. The videos produced by the creators are open to the public. The public can evaluate the video according to their preferences, or reward the creators. Weibo, Douyin, and Bilibili enable users to quickly access a wide array of the latest societal information and express their attitudes and opinions. Individuals on these platforms primarily establish weaker interpersonal connections. The survey collected data on participants’ frequency of social media usage, their perceived tie strength with social connections on each platform, and their self-reported experiences of being targeted by cyberbullying behaviors on these platforms.

## 3 Materials and methods

### 3.1 Procedure

This survey was conducted online through a Chinese investigation website^1^ from March 19 to March 29, 2023. We employed simple random sampling to collect data. The participants were primarily from Guangdong, Shandong, and Hebei provinces in China.

Before the formal survey, we conducted a pre-survey to estimate the required sample size and identify issues in the questionnaire. In the implementation stage of the pre-survey, according to the principle of randomness, we selected a university in Panyu District in Guangzhou. A total of 200 samples were collected for the pre-survey.

The planned sample size for the formal survey is 1,000 participants, considering that most regions in China have a robust internet infrastructure and a large number of internet users. In order to reduce the difficulty of sampling, we sampled the respondents according to the quota of university students and social members in advance. Additionally, considering that participants and victims of cyberbullying incidents on social media are primarily from the younger generation (ages 16–22), the sample size for this age group comprises 70% of the total sample, while other age groups account for 30%.

All participants were informed about the strict confidentiality of their responses before they filled out the survey, which included questions about demographics, the Chinese version of the Social Network Site Intensity Scale (Sun et al., 2016) and the Cyberbullying Inventory for College Students (CICS) (Tang et al., 2022). Each participant then provided an online informed consent form, acknowledging the voluntary nature of their participation. They were free to withdraw from the survey at any point. Participants were instructed to complete the questionnaire based on their actual experiences. Upon completion, participants received their compensation within 1 week.

### 3.2 Participants

A total of 1,036 participants completed the questionnaires, recruited from diverse cities across China. To maintain data quality and reliability, we set up five attention check questions and excluded participants who failed more than two of the five attention checks (*n* = 96). Moreover, we excluded participants whose response times exceeded or fell below three standard deviations from the mean (*n* = 114). We eliminated duplicate submissions from identical IP addresses (*n* = 13). Only one submission from each IP address that passed the attention and response time checks was retained. The application of the exclusion criteria resulted in a final sample of 813 participants, preserving approximately 78.47% of the initially collected data. Among the valid respondents, there were 315 males and 498 females, ranging in age from 16 to 49 years (*M* = 20.06, SD = 2.30). Participants had varying educational backgrounds and socioeconomic statuses: 3.5% were senior high school students or below, 1.6% were junior college students, 92.6% were undergraduate students, and 2.2% were master’s and doctoral students. All participants reported no history of physical or mental illness and no long-term dependence on psychotropic medications. The sample is close to being representative of the population under the study. Descriptive statistics of participants’ demographic information are presented in Table 1.

**Table 1:** Descriptive statistics for participants’ demographic information.

Factor	*n*	%
Gender		
Male	315	38.7%
Female	498	61.3%
One-child situation		
Only child	247	30.4%
Non-only child	566	69.6%
Age		
16–20 years old	580	71.3%
21–25 years old	217	26.7%
26–30 years old	11	0.8%
30 years old or above	5	1.2%
The education level of the participants		
Junior high school or lower	2	0.2%
High school/Technical school	27	3.3%
Associate degree	13	1.6%
Bachelor’s degree	753	92.6%
Master’s degree or above	18	2.2%
The education level of the participants’ father		
Junior high school or lower	353	43.4%
High school/Technical school	204	25.1%
Associate degree	124	15.3%
Bachelor’s degree	118	14.5%
Master’s degree or above	14	1.7%
The education level of the participant’s mother		
Junior high school or lower	393	48.3%
High school/Technical school	213	26.2%
Associate degree	92	11.3%
Bachelor’s degree	101	12.4%
Master’s degree or above	14	1.7%
Monthly household income		
Less than ¥2000	15	1.8
¥2000–¥5,000	141	17.3%
¥5,000–¥10,000	289	35.5%
¥10,000–¥20,000	249	30.6%
More than ¥20,000	119	14.6%

*N = 813.*

### 3.3 Measures

Cyberbullying is similar across social media platforms. Regarding measured indicators, cyberbullying incidents in China and various other countries are influenced by demographic factors (e.g., gender, age, education, and family wealth) as well as the intensity of users’ engagement with social media. In addition, the characteristics of cyberbullying exhibit a degree of universality. For example, the forms of cyberbullying in both China and the West are perpetrated online, and the content of bullying includes teasing others, fabricating rumors about others, invading others’ privacy, and sexual harassment. Therefore, the survey included questions about demographics, the Chinese version of the Social Network Site Intensity Scale (Sun et al., 2016), and the Cyberbullying Inventory for College Students (CICS) (Tang et al., 2022). Demographic information included gender, age, educational level, one-child status, and socioeconomic status. Socioeconomic status refers to the economic and social standing of an individual or family within society, typically measured by factors such as monthly household income, parental education level, and parental occupation. Due to the difficulty of quantifying different occupations, socioeconomic status in this study was measured by income and education level. Socioeconomic status was calculated as follows: monthly household income + parental education level. Participants rated their monthly household income on a 5-point Likert scale, from 1 (less than ¥2,000) to 5 (more than ¥20,000). They also rated their parental education level on a 5-point Likert scale, from 1 (junior high school or lower) to 5 (master’s degree or above). However, to better measure cyberbullying on Chinese social media platforms, we adapted the measurement indicators to the local context. In addition, when compiling the questionnaire, we conveyed the research content to participants in a manner more aligned with the Chinese context. The items in the questionnaire are presented in Table 2, while Table 3 shows the reliability of all measurements.

**Table 2:** The items in questionnaire scale.

Questionnaire	Variables	Items
The Chinese Version of Social Network Site Intensity Scale	U1	How much time do you spend on this media on average per day?
	U2	This social media app is part of my daily routine.
	U3	I would be proud to tell people that I use this social media.
	U4	Using this social media has become a daily habit for me.
	U5	When I do not log in to social media for a period, I feel disconnected from the outside world.
	U6	I feel like I’m part of this social media community.
	U7	I would be sorry if the social media no longer exists.
The Chinese Version of Cyberbullying Inventory for College Students	CB1	I threaten others through this social media.
	CB2	I harass people with sexual (pornographic) messages.
	CB3	I spread rumors about others.
	CB4	I impersonate others.
	CB5	I make fun of others.
	CB6	I insult others.
	CB7	I claim to have information that could embarrass someone.
	CB8	I expose other people’s private information
	CB9	I use (display) another person’s photo (video) without permission.
	BC1	Someone threatened me through this social media.
	BC2	Someone harassed me with sexual (pornographic) messages.
	BC3	Someone spread a rumor about me.
	BC4	Someone impersonated me.
	BC5	Someone made fun of me.
	BC6	Someone insulted me.
	BC7	Someone claims to have information that could embarrass me.
	BC8	Someone exposed my private information.
	BC9	Someone is using my photo (video) without permission.

**Table 3:** Reliability of the measurements.

Platform	Measurements	Reliability (Cronbach’s alpha)
WeChat	The intensity of social network site usage	0.788
	Cyberbullying	0.875
	Being cyberbullied	0.907
Weibo	The intensity of social network site usage	0.934
	Cyberbullying	0.907
	Being cyberbullied	0.943
Douyin	The intensity of social network site usage	0.950
	Cyberbullying	0.922
	Being cyberbullied	0.945
Bilibili	The intensity of social network site usage	0.874
	Cyberbullying	0.942
	Being cyberbullied	0.968

Cronbach’s alpha > 0.7, indicating high reliability.

#### 3.3.1 The Chinese version of social network site intensity scale

The Chinese version of the Social Network Site Intensity Scale (Sun et al., 2016) was used to measure the intensity of participants’ social network site usage. The questionnaire was originally developed by Ellison et al. (2007) and translated into Chinese by Sun et al. (2016), demonstrating good reliability and validity. The construct comprised the following items: U1. How many friends do you have on this social media. U2. How much time do you spend on this media on average per day. U3. This social media app is part of my daily routine. U4. I would be proud to tell people that I use this social media. U5. Using this social media has become a daily habit for me. U6. When I do not log in to social media for a period, I feel disconnected from the outside world. U7. I feel like I’m part of this social media community. U8. I would be sorry if the social media no longer exists.

The first two questions (U1 and U2) were used to measure the number of friends on social network sites and the average daily usage time of an individual, respectively. The remaining six items (U3 to U8) were scored on a 5-point Likert scale (1 “very inconsistent,” 5 “very consistent”) to measure the intensity of the emotional connection between the individual and social networking sites, as well as the degree of integration of social networking sites into their life.

However, since the survey subjects in this study include Bilibili, Douyin, and Weibo, it was difficult to calculate the number of friends that individuals had on these platforms. Therefore, the first item (U1) was removed from the measurement scale. Participants were asked to rate their average daily usage time of social media on a 6-point Likert scale, ranging from 1 (less than 10 min) to 6 (more than 3 h). The higher the score, the more time they spend on social media. They were asked to rate the intensity of the emotional connection between themselves and social media, as well as the degree of integration of social networking sites into their lives on a 5-point Likert scale, ranging from 1 (strongly disagree) to 5 (strongly agree). The higher the score, the more integrated social media is in their lives. On this basis, the questionnaire was adapted for four social media platforms: WeChat, Weibo, Douyin, and Bilibili, with users measured on these four platforms, respectively. The Cronbach’s *α* coefficients for the social network site usage scale were 0.815 for WeChat, 0.934 for Weibo, 0.952 for Douyin, and 0.878 for Bilibili, indicating good reliability.

The strength of a tie can be described as a combination of “the amount of time, emotional intensity, intimacy (mutual confiding), and reciprocal services that characterize the tie” (Granovetter, 1973). Based on these criteria, a range of tie strengths can be identified. However, the user’s social media usage itself includes the time spent on social media, the emotional intensity of the user’s investment in social media, and the trust and help the user receives from social media. Therefore, we can measure the intensity of the association between users and social media through the Chinese Version of Social Network Site Intensity Scale. Participants’ social media usage intensity index was calculated by summing the scores of the seven items answered by the participants.

#### 3.3.2 The Chinese version of cyberbullying inventory for college students

The Chinese version of the Cyberbullying Inventory for College Students (CICS) (Tang et al., 2022) was used to measure individuals’ experiences of cyberbullying on social media. The questionnaire was originally developed by Francisco et al. (2015) and translated into Chinese by Tang et al. (2022), demonstrating good reliability and validity. It contains two dimensions: cyberbullying and being cyberbullied. Thus, the inventory was used to measure individuals’ experiences of cyberbullying on social media. The cyberbullying construct comprised the following nine items: CB1. I threaten others through this social media. CB2. I harass people with sexual (pornographic) messages. CB3. I spread rumors about others. CB4. I impersonate others. CB5. I make fun of others. CB6. I insult others. CB7. I claim to have information that could embarrass someone. CB8. I expose other people’s private information. CB9. I use (display) another person’s photo (video) without permission.

The being cyberbullied construct comprised the following nine items: BC1. Someone threatened me through this social media. BC2. Someone harassed me with sexual (pornographic) messages. BC3. Someone spread a rumor about me. BC4. Someone impersonated me. BC5. Someone made fun of me. BC6. Someone insulted me. BC7. Someone claims to have information that could embarrass me. BC8. Someone exposed my private information. BC9. Someone is using my photo (video) without permission.

Participants were asked to rate their engagement in cyberbullying and the degree to which they have experienced cyberbullying on a 5-point Likert scale, ranging from 1 (never) to 5 (many times a week). The higher the scores, the more the individuals engage in or experience cyberbullying in their daily lives. Based on the four platforms in this study (WeChat, Weibo, Douyin, and Bilibili), the questionnaire was adapted to measure both cyberbullying and being cyberbullied on these four platforms. The Cronbach’s *α* coefficients for the cyberbullying subscales were 0.875 for WeChat, 0.907 for Weibo, 0.922 for Douyin, and 0.942 for Bilibili. The Cronbach’s α coefficients for the being cyberbullied subscales were 0.907 for WeChat, 0.943 for Weibo, 0.945 for Douyin, and 0.968 for Bilibili, indicating good reliability of the scales.

### 3.4 Data analysis

The independent variables were media usage, cyberbullying, and being cyberbullied, while the moderating variable was tie strength. The dependent variables included gender, age, only-child status, self-education level, father’s education level, mother’s education level, and socioeconomic status.

Pearson correlation analysis was performed to measure the correlations between all measured variables. A series of hierarchical regression analyses was used to examine the effects of the independent variables on the dependent variables. In the first step, controlled variables (i.e., gender, age, only-child status, education level, socioeconomic status) were included in the analysis. The independent variables were added to each regression model, allowing for the examination of their unique contributions to the dependent variables. Moderation analyses were conducted using the PROCESS macro version 4.1, specifically model 1, to examine the moderation models (Hayes, 2022). Controlled variables were included as covariates in each moderation model to account for their potential influence of 5,000 and the Confidence Interval (CI) of 95% were employed in the PROCESS analyses.

The average tie strength between users and the platforms is as follows: WeChat (*M* = 55.06, SD = 8.93), Bilibili (*M* = 42.61, SD = 13.47), Weibo (*M* = 33.16, SD = 16.14), and Douyin (*M* = 32.51, SD = 17.69).

We conducted a repeated measures ANOVA to assess the intensity of users’ connections with four social media platforms. Mauchly’s test indicated that the assumption of sphericity had been violated, χ^2^(5) = 252.641, *p* < 0.001. Therefore, degrees of freedom were corrected using Greenhouse–Geisser estimates of sphericity (0.613). The results indicated significant differences in the platforms’ strong and weak tie attributes. The results were presented in Table 4.

**Table 4:** The results of Mauchly’s test of sphericity.

Within subjects effect	Mauchly’s *W*	Approx. chi-square	df	*p*	Epsilon^a^		
					Greenhouse–Geisser	Huynh–Feldt	Lower-bound
Media usage	0.732	252.641	5	<0.001	0.836	0.839	0.333

*N = 813. Within subjects design is media usage. aMeans the degrees of freedom for the averaged tests of significance.*

Pairwise comparisons were conducted using the Bonferroni method. Statistical significance was set at a significance level of *p* < 0.05, and two-tailed hypothesis testing was employed with a 95% confidence interval.

The results of the pairwise comparative analysis show that the tie strength between users and WeChat is significantly different from that between users and Weibo (*p* < 0.001), Douyin (*p* < 0.001), and Bilibili (*p* < 0.001). Among these, the tie strength between users and Bilibili is significantly different from that between users and Weibo (*p* < 0.001) as well as from that between users and Douyin (*p* < 0.001). However, there is no significant difference in the tie strength between users and Weibo and between users and Douyin (*p* = 0.323). The results were presented in Table 5.

**Table 5:** The results of the pairwise comparisons for the intensity of users’ usage on the four platforms.

(I) Media usage	(J) Media usage	MD (I-J)	SE	*p*	95% CI^a^	
					LLCI	ULCI
WeChat	Weibo	21.904^*^	0.562	<0.001	20.418	23.391
	Douyin	22.550^*^	0.625	<0.001	20.897	24.202
	Bilibili	12.454^*^	0.496	<0.001	11.143	13.765
Weibo	WeChat	−21.904^*^	0.562	<0.001	−23.391	−20.418
	Douyin	0.646	0.653	1.000	−1.082	2.374
	Bilibili	−9.450^*^	0.629	<0.001	−11.114	−7.787
Douyin	WeChat	−22.550^*^	0.625	<0.001	−24.202	−20.897
	Weibo	−0.646	0.653	1.000	−2.374	1.082
	Bilibili	−10.096^*^	0.782	<0.001	−12.165	−8.027
Bilibili	WeChat	−12.454^*^	0.496	<0.001	−13.765	−11.143
	Weibo	9.450^*^	0.629	<0.001	7.787	11.114
	Douyin	10.096^*^	0.782	<0.001	8.027	12.165

*N* = 813. *Means difference is significant at the 0.05 level. ^a^Means adjustment for multiple comparisons is Bonferroni. MD is mean difference. SD is the standard deviation.

Therefore, the order of tie strength between users and the four platforms is as follows: WeChat > Bilibili > Weibo > Douyin. Among them, the tie strength of WeChat is significantly higher than that of the other three platforms. Thus, WeChat represents a strong tie, while Bilibili is a relatively weak tie, and both Weibo and Douyin are considered weak ties.

The platforms were encoded as dummy variables based on their tie strength properties (0 = weak-tie, 1 = strong-tie). Weibo, Douyin, and Bilibili were coded as 0, while WeChat was coded as 1. To address the potential impact on the accuracy of group 0 in model predictions, a new dummy variable was created by adding 1 to the original codes (1 = weak-tie, 2 = strong-tie).

## 4 Results

### 4.1 Descriptive and correlation analyses

The descriptive statistics (e.g., mean and standard deviation) and correlation matrix for all variables are presented in Table 6. As anticipated, tie strength showed a significant positive association with cyberbullying, being cyberbullied, and media usage (all |*r*| *s* ≥ 0.12, *p* s < 0.001). Moreover, media usage showed a significant positive association with cyberbullying, being cyberbullied, and tie strength (all |*r*| *s* ≥ 0.11, all *p* s < 0.001). Notably, cyberbullying showed a significant positive association with being cyberbullied (*r* = 0.787, *p* < 0.001). These relationships among the variables provide support for the subsequent hypothesis testing.

**Table 6:** Descriptive statistics and bivariate correlation.

Variable	M ± SD	1	2	3	4	5	6	7	8	9	10
1. Gender	1.61 ± 0.49	—									
2. Age	20.06 ± 2.30	−0.009	—								
3. Only-child status	1.70 ± 0.46	0.051**	0.024	—							
4. Self-education level	3.93 ± 0.43	−0.008	0.72***	0.008	—						
5. Father’s education level	2.06 ± 1.15	0.097***	−0.099***	−0.352***	−0.041*	—					
6. Mother’s education level	1.93 ± 1.12	0.124***	−0.135***	−0.356***	0.005	0.742***	—				
7. Socioeconomic status	0.00 ± 0.11	0.096***	−0.104***	−0.352***	−0.005	0.860***	0.875***	—			
8. Cyberbullying	9.73 ± 2.85	−0.076***	0.032	−0.064***	−0.74***	−0.004	−0.009	−0.013	—		
9. Being cyberbullyied	9.68 ± 2.93	−0.069***	0.019	−0.077***	−0.108***	−0.009	−0.011	−0.001	0.787***	—	
10. Tie strength	1.25 ± 0.43	<0.001	<0.001	<0.001	<0.001	<0.001	<0.001	<0.001	0.127***	0.118***	—
11. Media usage	40.83 ± 17.09	0.119***	0.006	0.007	−0.014	0.019	0.040*	0.045*	0.119***	0.117***	0.481***

*N* = 813. *p < 0.05, **p < 0.001, ***p < 0.001.

### 4.2 Linear-regression analysis

Multiple linear regression is a statistical technique used to establish a regression equation that quantifies the linear relationship between dependent and independent variables. To understand the relationship between users’ media usage, tie strengths, and demographic variables, Model 1 treated media usage as the dependent variable. Tie strength, gender, age, one-child status, education level, and socioeconomic status were included as independent variables due to their potential effects on users’ media usage. Specifically, prior scholars have identified that users’ various intentions and behaviors—such as media usage—could be affected by gender (Hunter and Taylor, 2019), age (Paulus et al., 2023), and education level (Jiang et al., 2023). A linear regression equation was constructed. The results are shown in Table 7.

**Table 7:** Regression analysis of users’ social media usage in the Model 1.

Dependent variable	Independent variables	*B*	SE	*β*	*t*	*p*	95% CI	
							LLCI	ULCI
Users’ social media usage	Intercept	10.053	3.519		2.857	0.004	3.154	16.952
	Tie strength	18.969	0.601	0.481	31.261	<0.001	17.791	20.148
	Gender	4.020	0.539	0.115	7.459	<0.001	2.963	5.077
	Age	0.085	0.114	0.011	0.748	0.454	−0.138	0.309
	Only-child status	0.555	0.607	0.015	0.915	0.360	−0.635	1.746
	Education level	−0.526	0.601	−0.013	−0.876	0.381	−1.705	0.652
	Socioeconomic status	0.278	0.114	0.040	2.434	0.015	0.054	0.503

*N = 813. SE is the standard error. LLCI is the lower level of confidence interval. ULCI is the upper level of the confidence interval.*

The results showed that tie strength (*β* = 0.481, *p* < 0.001), gender (*β* = 0.115, *p* < 0.001), and socioeconomic status (*β* = 0.040, *p* < 0.05) had significant positive effects on social media use behavior. In contrast, age (*β* = 0.011, *p* > 0.05), one-child status (*β* = 0.015, *p* > 0.05), and education level (*β* = −0.013, *p* > 0.05) did not have significant effects on individual social media usage.

To understand the relationship between users’ cyberbullying, tie strength, and demographic variables, Model 2 treated cyberbullying behavior as the dependent variable. It included eight factors as independent variables: tie strength, media usage, the interaction between tie strength and media usage, gender, age, one-child status, education level, and socioeconomic status. A linear regression equation was constructed. The results are shown in Table 8.

**Table 8:** Regression analysis of cyberbullying Model 2.

Dependent variable	Independent variables	*B*	SE	*β*	*t*	*p*	95% CI	
							LLCI	ULCI
Cyberbullying	Intercept	9.022	0.952		9.474	<0.001	7.155	10.889
	Tie strength	2.370	0.629	0.360	3.767	<0.001	1.136	3.604
	Media usage	0.052	0.013	0.311	4.007	<0.001	0.027	0.077
	Interactive items between tie strength and media usage	−0.034	0.012	−0.433	−2.953	0.003	−0.057	−0.011
	Gender	−0.460	0.102	−0.079	−4.490	<0.001	−0.661	−0.259
	Age	0.043	0.022	0.035	2.023	0.043	0.001	0.086
	Only-child status	−0.440	0.114	−0.071	−3.852	<0.001	−0.665	−0.216
	Education level	−0.483	0.113	−0.074	−4.262	<0.001	−0.705	−0.261
	Socioeconomic status	−0.036	0.022	−0.031	−1.671	0.095	−0.078	0.006

*N = 813. SE is the standard error. LLCI is the lower level of confidence interval. ULCI is the upper level of the confidence interval.*

The results showed that tie strength (*β* = 0.36, *p* < 0.001), media usage (*β* = 0.311, *p* < 0.001), and age (*β* = 0.035, *p* < 0.05) had significant positive effects on cyberbullying. Conversely, gender (*β* = −0.079, *p* < 0.001), the interaction between tie strength and media usage (*β* = −0.433, *p* < 0.05), one-child status (*β* = −0.071, *p* < 0.001), and education level (*β* = −0.074, *p* < 0.001) had significant negative effects on cyberbullying. Socioeconomic status had no significant effect on cyberbullying.

To understand the relationship between users’ exposure to cyberbullying, tie strength, and demographic variables, Model 3 treated users’ exposure to cyberbullying as the dependent variable. It included eight factors as independent variables: tie strength, media usage, the interaction between tie strength and media usage, gender, age, one-child status, education level, and socioeconomic status. A linear regression equation was constructed. The results are shown in Table 9.

**Table 9:** Regression analysis of being cyberbullied in the Model 3.

Dependent variable	Independent variables	*B*	SE	*β*	*t*	*p*	95% CI	
							LLCI	ULCI
Being cyberbullied	Intercept	10.784	0.977		11.034	<0.001	8.868	12.700
	Tie strength	1.855	0.646	0.274	2.873	0.004	0.589	3.121
	Media usage	0.043	0.013	0.249	3.212	0.001	0.017	0.069
	Interactive items between tie strength and media usage	−0.025	0.012	−0.311	−2.129	0.033	−0.048	−0.002
	Gender	−0.437	0.105	−0.073	−4.153	<0.001	−0.643	−0.230
	Age	0.033	0.022	0.026	1.487	0.137	−0.010	0.076
	Only-child status	−0.523	0.117	−0.082	−4.456	<0.001	−0.753	−0.293
	Education level	−0.727	0.116	−0.108	−6.256	<0.001	−0.955	−0.499
	Socioeconomic status	−0.029	0.022	−0.024	−1.294	0.196	−0.072	0.015

*N = 813. SE is the standard error. LLCI is the lower level of confidence interval. ULCI is the upper level of the confidence interval.*

The results showed that tie strength (*β* = 0.274, *p* < 0.05) and media usage (*β* = 0.249, *p* < 0.05) had significant positive impacts on users’ exposure to cyberbullying. In contrast, gender (*β* = −0.073, *p* < 0.001), one-child status (*β* = −0.082, *p* < 0.001), education level (*β* = −0.108, *p* < 0.001), and the interaction between tie strength and media usage (*β* = −0.311, *p* < 0.05) had significant negative effects on users’ exposure to cyberbullying. Age and socioeconomic status had no significant effects on users’ exposure to cyberbullying.

### 4.3 Interaction effect

The moderating effect refers to a phenomenon in research where a specific variable (the moderator) influences the relationships between two variables. In other words, the moderator can alter the strength or direction of the relationship between the independent variable and the dependent variable. This concept aids researchers in understanding how the relationships between variables may vary across different conditions, thereby providing a more nuanced analysis and interpretation.

**Figure 1:**
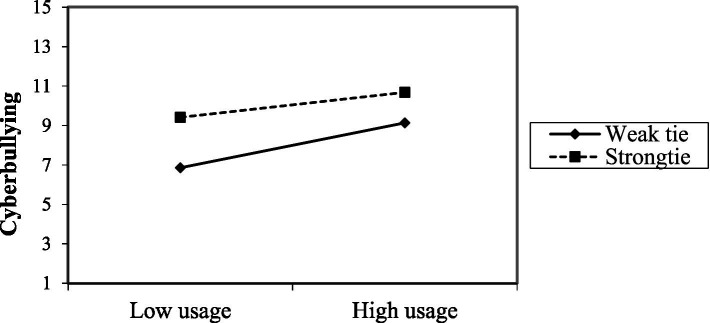
Interactive effects of media usage and cyberbullying.

To understand whether the relationship between media usage and cyberbullying was influenced by tie strength, Model 1 from PROCESS version 4.1, developed by Hayes (2022), was used to conduct an interaction effect verification. The results indicated that the interaction effect was significant (*p* = 0.003). The results are shown in Tables 10, 11 and Figure 1.

**Table 10:** Outcomes of interaction between media usage and cyberbullying.

Variables	Coeff	SE	*t*	*p*	LLCI	ULCI
Constant	9.022	0.952	9.474	<0.001	7.155	10.889
Social media usage	0.052	0.013	4.007	<0.001	0.027	0.077
Tie strength	2.370	0.629	3.767	<0.001	1.136	3.604
Interactive items between tie strength and media usage	−0.034	0.012	−2.954	0.003	−0.057	−0.011
Gender	−0.460	0.102	−4.490	<0.001	−0.660	−0.259
Age	0.044	0.022	2.023	0.043	0.001	0.086
Only-child status	−0.441	0.114	−3.852	<0.001	−0.665	−0.216
Education level	−0.483	0.113	−4.262	<0.001	−0.705	−0.261
Socioeconomic status	−0.036	0.022	−1.671	0.095	−0.078	0.006

*N* = 813. Outcome variable is Cyberbullying. Tie strength was dummy coded (1 = weak tie, 2 = strong tie). Coeff is the coefficient. SE is the standard error. LLCI is the lower level of confidence interval. ULCI is the upper level of the confidence interval.

**Table 11:** Conditional effects of the focal predictor at values of the moderator(s).

Tie strength	Effect	SE	*t*	*p*	LLCI	ULCI
Weak tie	0.018	0.004	5.177	<0.001	0.011	0.025
Strong tie	−0.016	0.011	−1.466	0.143	−0.038	0.006

Focal predictor is media usage. Moderating variable is tie strength. Outcome variable is cyberbullying. LLCI is the lower level of confidence interval. ULCI is the upper level of the confidence interval. SE is the standard error.

Similarly, when utilizing PROCESS version 4.1 to examine whether the association between media usage and being cyberbullied was influenced by tie strength, it was found that the interaction effect was significant (*p* < 0.001). The results are shown in Table 12.

**Table 12:** Outcomes of interaction between media usage and being cyberbullied.

Variables	Coeff	SE	*t*	*p*	LLCI	ULCI
Constant	10.784	0.977	11.034	<0.001	8.868	12.700
Media usage	0.043	0.013	3.212	0.001	0.017	0.069
Tie strength	1.855	0.646	2.873	0.004	0.589	3.121
Interactive items between tie strength and media usage	−0.025	0.012	−2.129	0.033	−0.048	−0.002
Gender	−0.437	0.105	−4.153	<0.001	−0.643	−0.231
Age	0.033	0.022	1.487	0.137	−0.010	0.076
Only-child status	−0.523	0.117	−4.456	<0.001	−0.753	−0.293
Education level	−0.727	0.116	−6.256	<0.001	−0.955	−0.499
Socioeconomic status	−0.029	0.022	−1.294	0.196	−0.072	0.015

*N* = 813. Outcome variable is Being cyberbullied. Tie strength was dummy coded (1 = weak tie, 2 = strong tie). Coeff is the coefficient. SE is the standard error. LLCI is the lower level of confidence interval. ULCI is the upper level of the confidence interval.

**Figure 2:**
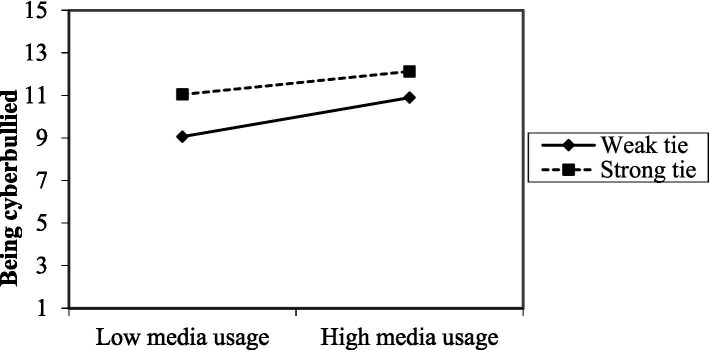
Interactive effects of media usage and being cyberbullied.

Based on the results of the conditional effect of the focal predictor at the value of the moderator, we found that a weak tie significantly influences the moderating effect of being cyberbullied (*p* < 0.001), while a strong tie has no significant effect on the moderating effect of cyberbullying (*p* = 0.500) (Figure 2 and Table 13).

**Table 13:** Conditional effects of the focal predictor at values of the moderator(s).

Tie strength	Effect	SE	*t*	*p*	LLCI	ULCI
Weak-tie	0.018	0.004	4.947	<0.001	0.011	0.025
Strong-tie	−0.008	0.011	−0.675	0.500	−0.030	0.015

Focal predictor is media usage. Moderating variable is tie strength. Outcome variable is being cyberbullied. LLCI is the lower level of confidence interval. ULCI is the upper level of the confidence interval. SE is the standard error.

## 5 Discussion

Cyberbullying is intrinsically linked to media use. This study examines the relationship between media usage and cyberbullying, focusing on the moderating role of tie strength across different platforms. We measured 813 participants’ media use and cyberbullying experiences across four Chinese platforms through an online survey. Results revealed that platforms with strong ties exhibited higher user engagement compared to those with weak ties. Increased media usage was associated with a higher likelihood of both perpetrating and experiencing cyberbullying. Tie strength appeared to moderate this association: on platforms with strong ties, increased usage was associated with a lower likelihood of cyberbullying involvement, whereas on platforms with weak ties, the opposite pattern was observed. These findings highlight the moderating role of tie strength in the association between media usage and cyberbullying, suggesting that the tie strength of media platforms significantly influences online behaviors. These insights can inform future research and interventions aimed at addressing cyberbullying.

The results of this investigation lend credence to Hypothesis 1, which states that the tie strength of social media is associated with the intensity of users’ media usage. That is, a higher intensity of media usage on strong-tie social media is associated with a lower intensity of usage on weak-tie social media. Consistent with the existing body of literature, this research shows that on social media, the tie strength of relationships is an important factor affecting users’ participation in social behaviors. The study by Bakhshi et al. (2014) also demonstrates that the tie strength of relationships can affect the information transfer and knowledge-sharing behaviors among users. These findings provide empirical evidence supporting the relationship between tie strength and users’ media usage, thereby contributing to the understanding of how social dynamics influence online behavior.

Furthermore, the research identified that the intensity of media usage is associated with cyberbullying, lending support to Hypothesis 3a. Specifically, the research found that the intensity of users’ media usage has a significant positive impact on their cyberbullying behaviors. A higher intensity of a user’s media usage is associated with a greater likelihood of engaging in cyberbullying. Conversely, the lower the intensity of a user’s media usage, the smaller the probability of engaging in cyberbullying. In addition, this study found that the intensity of users’ media usage has a positive effect on their exposure to cyberbullying, corroborating Hypothesis 3b. Existing literature indicates that excessive internet use is associated with various negative outcomes, including physical health problems, poor academic performance, issues with family relationships, poorer social connections, emotional symptoms (e.g., anxiety and unhappiness), and peer relationship problems (e.g., having few friends and being bullied) (Liu et al., 2024; Shi et al., 2024; Joshi et al., 2023). Additionally, Carli et al. (2012) found an association between excessive internet use and various mental health issues, including depression, anxiety, attention deficit and hyperactivity disorder (ADHD), and aggression, in children and young adults. Cyberbullying involves a complex set of correlations, and its mitigation requires long-term cooperation from platforms, society, and users. Given these findings, enhancing users’ digital literacy is essential for mitigating cyberbullying and fostering a healthier internet environment.

In addition, this study found that tie strength is significantly associated with both cyberbullying and being cyberbullied. Specifically, users in strong-tie social media networks are more likely to engage in cyberbullying and to be victims of cyberbullying compared to users in weak-tie networks. These findings bolster support for Hypotheses 2a and 2b, which suggest that the nature of social connections plays a critical role in understanding cyberbullying dynamics and contribute to the broader discourse on online behavior.

Moreover, by analyzing the interaction between tie strength and media usage on cyberbullying, this study found that the association between media usage and cyberbullying was moderated by tie strength. Specifically, this study found that: (1) the influence of media usage on cyberbullying was significant only when the tie strength of social media was weak. In other words, in weak-tie social media, a higher intensity of a user’s media usage is associated with a greater likelihood of engaging in cyberbullying behaviors. In contrast, when users engage with strong-tie social media, the intensity of their media usage does not significantly affect their cyberbullying behaviors. (2) The association between media usage and being a victim of cyberbullying is similarly moderated by tie strength, being significant only in weak-tie social media networks. In other words, in weak-tie social media networks, a higher intensity of a user’s media usage is associated with a greater probability of that user being a victim of cyberbullying. However, when users engage with strong-tie social media, the intensity of their media usage does not affect their likelihood of being cyberbullied.

In conclusion, this study found a significant interaction between tie strength theory and medium theory, which jointly explains the impact of users’ social media usage on cyberbullying behaviors. Specifically, in strong-tie social media networks, users’ intensity of media usage is associated with both their participation in cyberbullying and their likelihood of becoming victims of cyberbullying. In contrast, medium theory provides a better understanding of users’ behaviors and experiences in weak-tie social media environments, where higher media usage intensity is associated with increased cyberbullying perpetration and victimization. Overall, these findings demonstrate the complex interplay between relationship strength and media usage in shaping cyberbullying dynamics. In weak-tie social media, users’ media usage is not associated with their participation in or experiences of cyberbullying, suggesting that medium theory may not effectively account for behaviors in this context. Although users’ media usage did not have a significant effect on cyberbullying behavior or on being cyberbullied in weak-tie social media, this does not mean that social media is irrelevant in this context. Factors such as individual characteristics, social environment, and interpersonal relationships may play a more significant role in shaping cyberbullying dynamics.

Weibo has established a five-step system for addressing online violence, which includes institutional constraints, technological identification, content moderation, function protection, and public education. However, social media platforms still lack comprehensive governance mechanisms that address the root causes of cyberbullying. Key areas for improvement include inadequate user qualification reviews, insufficient efforts to cultivate positive community atmospheres, and the absence of effective tracking and educational initiatives.

To address these gaps, platforms should implement a more comprehensive seven-step governance framework. This framework should include user qualification audits, initiatives to foster a positive community atmosphere, advanced technological identification, robust reward and punishment systems, functional guarantees, network credit systems, and ongoing tracking and educational programs: (1) User qualification audits. Platforms can draw on Bilibili’s approach, which mandates that new users achieve a minimum score of 60 on an exam to access all features. Social media platforms could implement assessment questions related to cyberbullying during user registration. This strategy would help users understand the platform’s culture and promote awareness of ‘civilized internet usage. Furthermore, the assessment results could yield valuable insights for user classification and cyberbullying management. (2) Initiatives to foster a positive community atmosphere. The homepage should regularly promote awareness of cyberbullying and anti-bullying measures to remind users to maintain a friendly environment. Additionally, the comments section should display prompts encouraging ‘civilized communication’ to help users think carefully before posting. Furthermore, platforms must refine their cyberbullying management regulations and monitor relevant terms and discussion groups to provide timely assistance to potential victims and warn perpetrators. (3) Advanced technological identification. Social media platforms should promptly track cyberbullying incidents by establishing a keyword corpus through opinion monitoring and utilizing long-tail keywords to identify relevant content. Given the rapid internet development, regular updates to the corpus and training of new models are essential. In addition to automated monitoring, emphasis should be placed on training human moderators, such as “content reviewers” and “bullying prevention specialists.” During content review, if a user’s content closely resembles entries in the ‘cyberbullying keyword database,’ a warning mechanism should restrict posting capabilities and display anti-cyberbullying messages to improve digital literacy. (4) Robust reward and punishment systems. Platforms should establish a reward mechanism to encourage users who identify and report cyberbullying, aiming to reduce the “diffusion of responsibility” and promote positive engagement among bystanders. Rewards may include increased account points, small monetary incentives, or tangible gifts. Additionally, the platform should impose corresponding penalties for varying levels of cyberbullying behavior based on community regulations and cybersecurity laws. (5) Functional guarantees. The platform should enhance support for cyberbullying victims by establishing a counseling section in collaboration with social psychological organizations to offer free counseling and courses. System notifications can be used to show humanistic care for victims. Additionally, the platform should assist victims in collecting evidence and improve product features, including adjusting direct messaging rules, comment policies, and do-not-disturb settings. (6) Network credit systems. The platform should take social responsibility by clearly defining cyberbullying in community rules. For banned or warned accounts, timely and transparent disclosure of information is essential through an official public area. This area should detail rule violations and penalties while concealing certain usernames, serving as a warning to users and fostering accountability. (7) Ongoing tracking and educational programs. For accounts that have previously violated rules, the platform should implement a “one-year follow-up education mechanism” to monitor and educate these users while verifying their eligibility. Users who repeatedly violate rules during this period will face disciplinary measures based on the severity of their violations. Accounts that successfully complete the assessment will have their permissions restored.

By implementing such a multifaceted governance model, social media platforms can work toward more effectively preventing, mitigating, and remediating the complex challenges of online violence and harassment.

## 6 Limitations and future directions

This study provides valuable insights into the impact of demographic factors and social media usage on the phenomenon of cyberbullying. However, the research has some notable limitations. For instance, the cross-sectional design and predominantly student-based sample may result in a somewhat one-sided perspective, which only enables us to infer correlations (but not causality) between media usage, cyberbullying, and tie strength, potentially overlooking the influence of differing backgrounds, motivations, age and usage patterns in workplace contexts. To gain a more comprehensive understanding, we would suggest that future studies could be extended to a wider population, including working professionals, and conduct longitudinal analyses of social media use in workplace settings to verify whether our findings are generalizable.

Furthermore, the study focused on four of China’s most prominent social media platforms. While these represent a significant user base, previous research has suggested that well-known products may influence user susceptibility to certain behaviors. Consequently, the applicability of the current findings to lesser-known social media environments remains uncertain. Future research should explore a broader range of platforms to gain a more holistic view of cyberbullying dynamics.

To understand cyberbullying in weak-tie social media, we need to look beyond medium theory and explore other theoretical frameworks that can better explain users’ behaviors and experiences in these environments. Follow-up studies should further investigate alternative factors, such as individual characteristics, social environments, and interpersonal relationships, that may have a more significant impact on cyberbullying dynamics within weak-tie social networks. This underscores the need for a more comprehensive and tailored approach to preventing and supervising cyberbullying that accounts for the distinct characteristics of different social media contexts.

Finally, given the rapidly evolving nature of social media and internet technologies, the research instruments utilized in this study may require periodic adjustments to remain relevant and to accurately capture emerging trends. Continuous refinement and adaptation of the research approach over time will be essential to sustaining the accuracy and relevance of such investigations.

## 7 Conclusion

This article highlights that previous studies largely overlooked the specific platforms involved in cyberbullying and did not differentiate how various platform characteristics influence user behavior. Moreover, tie strength theory was not utilized in the analysis. This study addresses these gaps by revealing the potential impact of personal characteristics on cyberbullying and confirming that social media with different tie strengths affects users’ behaviors in distinct ways, thereby enhancing the application of tie strength theory. It also suggests that the effectiveness of medium theory in explaining media’s influence on user behavior may depend on the media’s characteristics.

## Data Availability

The raw data supporting the conclusions of this article will be made available by the authors, without undue reservation.

## Footnotes

^1^ http://www.sojump.com
